# Irritable Testis

**Published:** 1878-10

**Authors:** 


Irritable Testis.—Despite the statement to that effect in a recent and very valuable work on genito-urinary diseases, I doubt if surgeons who have treated many cases of this affection will concur in denominating it a species of neuralgia of the gland. The excessive sensitiveness of the organ is shown by the agony which the most careful contact, or the most insignificant shock will induce. Sometimes this morbid sensitiveness exists all over the gland; again, it is only one spot that is affected. In the case of the patient whose history I desire to allude to in this connection, the whole left testicle at first seemed to be morbidly irritable and hypersensitive; on more careful manipulation it was found that the organ could be handled without exciting pain, provided the observer was careful to avoid touching the globus major, part of the body of the epididymis and a small section of the gland adjacent to those parts. When the patient walked along the street, and a fold of his clothing came against the above enumerated parts, the agony excited would be almost unendurable. His sufferings came on gradually. One morning in January, 1877, this patient noticed that his left testicle seemed unusually sensitive. It gradually grew worse, and a month afterward he was in much the same condition as when he came under my observation, in August, 1877. At that time he wore an anxious, worn and broken appearance, which would attract attention anywhere, and he informed me that for several weeks he had been utterly incapacitated for either mental or physical labor. He could ascribe no cause for the development of the disease; he assured me that he was a man of chaste habits and moral life, and that he neither abused his genital organs nor subjected himself to such temptations as would excite his animal propensities. He mentioned falling down stairs in December, 1876, and striking the lower part of his abdomen against a footstool, but did not think the accident could have contributed to the development of his disease. The testicle was neither swollen nor unduly congested, and there were no local indications of disease other than the great sensitiveness of the parts already enumerated. The local treatment prescribed at first did the patient no good, and when he came to town for further advice, in October, he was greatly discouraged. He then told me that he had been under the care of several physicians since January; that none of them had done him any good; in fact, he believed he was worse at that time than he had been for months. One particular symptom, he remarked, of late development, caused him an amount of distress that was simply inconceivable. For instance, when gas was generated in his bowels, and passed into the large intestine, if he made an attempt to prevent its immediate discharge by contracting the muscles of the rectum, the same sensation was felt in the testicle as if that organ had been suddenly and forcibly compressed. On this account he had latterly avoided all society.

My attention having been attracted to this last symptom, more, perhaps, from my inability to explain it, than from any other reason, I finally determined to make a rectal examination. The patient readily assented to this, and bore all my manipulations unflinchingly until I endeavored to test the sensibility of the parts above the upper margin of the base of the prostate; as soon as I brought my finger in contact with the anterior wall of the rectum the patient screamed with pain, and I had to conclude my investigation at once. This distress was located in the left testicle. On my asking the question directly, he said he believed that there was pain at the base of the bladder, which radiated down the left spermatic cord, but his answer was so manifestly suggested by my question that I placed no reliance upon it. After thinking the matter over, I explained to him my reasons for believing that counter-irritation over the base of the prostate and the region of the vesiculæ seminales would do him good, and obtained his consent to that plan of treatment. He had such a horror of a digital exploration of that region that I placed him in the knee-chest position necessary for the introduction of Sims' speculum, and in lieu of the latter, used Kramer's ear speculum. Upon separating the blades of the latter when introduced sufficiently far to include the internal sphincter, I found the anterior wall of the rectum completely distended, and had no trouble in applying vesicating collodion over the whole region corresponding with the prostate and vesiculæ seminales. After keeping the parts exposed to the air until the collodion dried, I withdrew the speculum, after placing in contact with the visicant a wad of cotton saturated with glycerine. In an hour after the application was made, the patient took passage on an Ohio river boat, for his home, near Cincinnati, promising to return in a week or ten days. At the expiration of the appointed time, I received a letter from this gentleman, in which he said that his progress had been "splendid," and that circumstances were such that he could not visit me before the first of December. During the first week of December I met him on one of the river boats, in compliance with the request he telegraphed me. He said that he noticed nothing unusual for several hours after I applied the collodion to the rectal aspect of his genito-urinary organs; then a sensation of smarting and burning was developed, and caused him great apprehension. This kept him uneasy for two days; accident then led him to discover that his testicle was no longer so sensitive as formerly. When his bowels moved, he suffered a little, but he experienced no pain in his testicle. In short, in a week from the day the application was made to the rectum the morbid sensibility of his testicles had completely subsided, and in July, 1878, he told me there had been no return of the trouble.

While one case proves but little, there can be no objection to quoting the foregoing as an illustration of the efficacy of counter-irritation in a case of irritable testis, in which the probabilities are that the disease was of traumatic origin. Far more important than the question of the cure of this case by local applications to the rectal aspect of the genito urinary organs, is the point illustrated by the method of treatment. The same principle which permits the surgeon to heal the diseases of the uterus under sight is equally available in the treatment of diseases of the lower bowel in both sexes, and bids fair to prove of great service in the prevention and cure of an important class of genito-urinary affections in men. Counter-irritation in the same way described in the foregoing case has relieved five patients with disease of the prostate gland during the past three months. The application of vesicating collodion has likewise proved efficacious in preventing epididymitis in two cases with urethritis, who were threatened with swelling of testicle a few weeks since.—*Medical and Surgical Reporter.*

